# Membrane Rafts: Portals for Viral Entry

**DOI:** 10.3389/fmicb.2021.631274

**Published:** 2021-02-04

**Authors:** Inés Ripa, Sabina Andreu, José Antonio López-Guerrero, Raquel Bello-Morales

**Affiliations:** ^1^Departamento de Biología Molecular, Universidad Autónoma de Madrid, Madrid, Spain; ^2^Centro de Biología Molecular Severo Ochoa, CSIC-UAM, Madrid, Spain

**Keywords:** cholesterol, viral entry, endocytosis, caveolae, raft

## Abstract

Membrane rafts are dynamic, small (10–200 nm) domains enriched with cholesterol and sphingolipids that compartmentalize cellular processes. Rafts participate in roles essential to the lifecycle of different viral families including virus entry, assembly and/or budding events. Rafts seem to participate in virus attachment and recruitment to the cell surface, as well as the endocytic and non-endocytic mechanisms some viruses use to enter host cells. In this review, we will introduce the specific role of rafts in viral entry and define cellular factors implied in the choice of one entry pathway over the others. Finally, we will summarize the most relevant information about raft participation in the entry process of enveloped and non-enveloped viruses.

## Introduction

“Membrane rafts” or “lipid rafts” are small, dynamic membrane domains enriched with cholesterol and sphingolipids present in the plasma membrane, as well as in intracellular membranes and extracellular vesicles. Membrane rafts have the ability to concentrate or segregate specific elements in order to regulate their interactions with other components. Rafts may induce conformational changes in resident proteins, affecting their activity (Sezgin et al., 2017). Because of this, lipid rafts are essential for maintenance of cellular functions such as signal transduction (Koyama-Honda et al., 2020), receptor activation (Shi and Ruan, 2020), intracellular lipid and protein trafficking (Ouweneel et al., 2020), spatial organization of the plasma membrane (van IJzendoorn et al., 2020), endocytosis (Nichols, 2003a) and extracellular vesicle formation (Skryabin et al., 2020). As a consequence of their broad involvement in cell physiology, lipid rafts play an important role in complex processes including immune response (Varshney et al., 2016), host–pathogen interaction (Bukrinsky et al., 2020), cancer development (Greenlee et al., 2020), and cardiovascular disorders (Das and Das, 2009).

**FIGURE 1:**
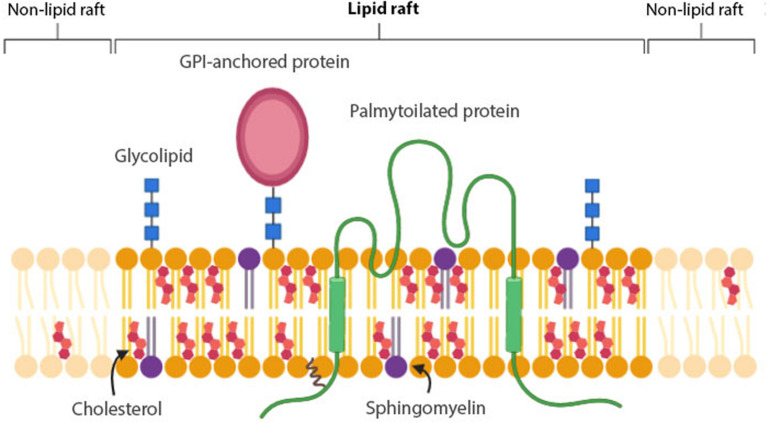
Membrane raft structure. Lipid rafts are composed of cholesterol, saturated phospholipids and sphingolipids, such as glycolipids and sphingomyelin (SM). GPI-anchored proteins and lipidated – especially palmitoylated- proteins have a higher affinity for lipid rafts than non-lipid rafts.

Regarding host–pathogen interactions, membrane rafts have been shown to play a role in viral life cycles, especially in processes like virus entry, assembly and/or budding (Chazal and Gerlier, 2003; Takahashi and Suzuki, 2009, 2011). Viruses are obligate intracellular parasites that must transport their genomes from infected cells to uninfected ones in order to initiate each new round of replication. To facilitate entry into cells, most viruses hijack cellular machinery, especially endocytic mechanisms. Only a few viruses are capable of directly penetrating the cell surface, crossing into the cytoplasm by fusing their envelope with the plasma membrane (Yamauchi and Helenius, 2013). Membrane rafts are implied in both endocytic mechanisms and viral entry via fusion (Figure 1). This review will focus on the involvement of rafts in the entry of enveloped (Table 1) and non-enveloped (Table 2) viruses, breaking down current studies in the field according to viral family.

**TABLE 1:** Entry of enveloped viruses mediated by membrane rafts.

Family	Virus	Role of membrane rafts in attachment and/or entry
*Coronaviridae*	Canine respiratory coronavirus (CRCoV)	Caveolae-mediated endocytosis (Szczepanski et al., 2018).
	Human coronavirus (HCoV) 229E	Caveolae-mediated endocytosis. Receptor aminopeptidase N (APN/CD13) on rafts (Nomura et al., 2004).
	Human coronavirus (HCov) OC43	Caveolae-mediated endocytosis (Owczarek et al., 2018).
	Infectious bronchitis virus (IBV)	Rafts for virus attachment (Guo et al., 2017).
	Murine hepatitis virus (MHV)	Raft-dependent fusion (Thorp and Gallagher, 2004; Choi et al., 2005).
	Porcine epidemic diarrhea virus (PEDV)	Caveolae-mediated endocytosis (Wei et al., 2020).
	Severe acute respiratory syndrome coronavirus (SARS-CoV)	Non-clathrin non-caveolae endocytosis (Wang et al., 2008). Receptor angiotensin-converting enzyme 2 (ACE2) on rafts (Glende et al., 2008; Lu et al., 2008).
	Severe acute respiratory syndrome coronavirus 2 (SARS-CoV-2)	Receptor ACE2 on rafts (Baglivo et al., 2020; Hoffmann et al., 2020; Wang H. et al., 2020).
	Transmissible gastroenteritis virus (TGEV)	Caveolae-mediated endocytosis (Wang J. et al., 2020)
*Filoviridae*	Ebola virus (EBOV)	Caveolae-mediated endocytosis (Empig and Goldsmith, 2002; Sanchez, 2007). Raft-dependent fusion in endosomal compartments (Freitas et al., 2011). Raft-dependent macropinocytosis (Jin et al., 2020).
	Marburg virus	Caveolae-mediated endocytosis (Empig and Goldsmith, 2002; Sanchez, 2007).
*Flaviviridae*	Classical Swine Fever Virus (CSFV) Shimen strain	Caveolae-mediated endocytosis (Ning et al., 2016; Zhang et al., 2018b).
	Dengue virus (DENV)	Receptors dendritic cell-specific ICAM 3-grabbing non-integrin (DC-SIGN) (Navarro-Sanchez et al., 2003; Tassaneetrithep et al., 2003; Cambi et al., 2004), heat shock proteins HSP90 and HPS70 (Reyes-del Valle et al., 2005) on rafts (Goodman et al., 2018).
	Human hepatitis C virus (HCV)	Rafts for virus attachment (Kapadia et al., 2007; Helle and Dubuisson, 2008; Voisset et al., 2008). Receptors CD81 tetraspanin (Soldaini et al., 2003; Cherukuri et al., 2004; Koutsoudakis et al., 2007), scavenger receptor B type I (SR-BI) (Scarselli et al., 2002; Rhainds et al., 2004), claudin-1 (Evans et al., 2007; Lynch et al., 2007) on rafts.
	Japanese encephalitis virus (JEV)	Caveolae-mediated endocytosis (Lee et al., 2008; Zhu et al., 2012a; Kalia et al., 2013). Rafts for virus attachment and cell signaling. Recruitment of the receptor HSP70 into rafts (Das et al., 2010; Zhu et al., 2012b).
	West Nile virus (WNV)	Raft-dependent entry (Medigeshi et al., 2008).
*Herpesviridae*	Equine herpesvirus-1 (EHV-1)	Caveolae-mediated endocytosis (Hasebe et al., 2009).
	Human herpesvirus type-6 (HHV-6)	Recruitment of the receptor CD46 into rafts. Association of viral glycoprotein Q1 with rafts (Tang et al., 2008; Tang and Mori, 2010).
	Kaposi’s sarcoma-associated herpesvirus (KSHV)	Raft-dependent macropinocytosis (Raghu et al., 2007; Veetti et al., 2010). Recruitment of the receptors α3β1 and αVβ3 integrins and amino acid transporter x-CT into rafts (Chakraborty et al., 2011; Veettil et al., 2014). Signaling amplification via tyrosine kinase Ephrin A2 (EphA2) into rafts (Chakraborty et al., 2012; Hahn et al., 2012).
	Herpes simplex virus type-1 (HSV-1)	Raft-dependent fusion (Weed and Nicola, 2017). Association of viral glycoprotein gB with rafts (Bender et al., 2003). Fusogenic activity of glycoprotein gH on rafts (Vitiello et al., 2015). Recruitment of the receptor nectin-1 into rafts. Cholesterol sensitive and dynamin-2- mediated endocytosis (Gianni et al., 2010; Gianni and Campadelli-Fiume, 2012).
	Pseudorabies virus (PRV)	Raft-dependent entry (Desplanques et al., 2008).
*Iridoviridae*	Infectious spleen and kidney necrosis virus (ISKNV)	Caveolae-mediated endocytosis (Guo et al., 2012)
	Tiger frog virus (TFV)	Caveolae-mediated endocytosis (Guo et al., 2011)
*Orthomyxoviridae*	Influenza A virus (IAV)	Raft-dependent endocytosis. Membrane rafts for virus multivalent binding to terminal sialic acid (SIA) (Verma et al., 2018).
*Paramyxoviridae*	Human metapneumovirus (hMPV)	Raft-dependent entry (Chen et al., 2019a)
	Newcastle disease virus (NDV)	Caveolae-mediated endocytosis (Cantín et al., 2007)
*Phenuiviridae*	Rift Valley fever virus (RVFV)	Caveolae-mediated endocytosis (Harmon et al., 2012).
*Poxviridae*	Vaccinia virus	Viral envelope proteins A14L, A17L and D8L on cell membrane rafts (Chung et al., 2005). Receptor vaccinia virus penetration factor (VPEF) on rafts (Huang et al., 2008). Recruitment of virus particles and the receptor CD98 into rafts (Izmailyan et al., 2012; Schroeder et al., 2012).
*Retroviridae*	Amphotropic murine leukemia virus (A-MLV)	Caveolae-mediated endocytosis. Receptor Pit- on rafts (Beer et al., 2005; Beer and Pedersen, 2006).
	Avian sarcoma and leukosis virus (ASLV)	Receptor GPI-anchored TVA (TVA800) on rafts (Narayan et al., 2003).
	Ecotropic murine leukemia virus (E-MLV)	Receptor CAT1 on rafts associated with caveolin (Lu and Silver, 2000).
	Human immunodeficiency virus (HIV)	Raft-dependent fusion in T lymphocytes (Yang et al., 2016, 2017; Molotkovsky et al., 2018). Receptor CD4 (Millán et al., 1999; Kozak et al., 2002) and co-receptor CCR5 on rafts (Popik et al., 2002). Recruitment of co-receptor CXCR4 into raft periphery (Popik et al., 2002; Kamiyama et al., 2009). “Pathway of HIV Endocytic Entry in Macrophages” (PHEEM): non-clathrin non-caveolae dynamin-dependent endocytosis which shares features with macropinocytosis (Carter et al., 2009, 2011; Gobeil et al., 2013). Dependence on raft CD4 location (Van Wilgenburg et al., 2014). Caveolae-mediated endocytosis in mucosal epithelial cells (Yasen et al., 2018).
	Human T lymphotropic virus 1 (HTLV-1)	Receptors glucose transporter 1 (GLUT-1) (Barnes et al., 2004; Kumar et al., 2004; Wielgosz et al., 2005), neuropilin-1 (Guirland et al., 2004; Ghez et al., 2006; Moretti et al., 2008; Salikhova et al., 2008) on rafts.

**TABLE 2:** Entry of non-enveloped virus mediated by membrane rafts.

Family	Virus	Role of membrane rafts in attachment and/or entry
*Adenoviridae*	Human adenovirus species C (HAdV-C)	Caveolae-mediated endocytosis (Colin et al., 2005; Rogée et al., 2007)
	Human adenovirus species D (HAdV-D)	Caveolae-mediated endocytosis (Yousuf et al., 2013).
*Papillomaviridae*	Human papillomavirus strain 31 (HPV-31)	Caveolae-mediated endocytosis (Bousarghin et al., 2003; Smith et al., 2007, 2008).
*Parvoviridae*	Adeno-associated virus 2 (AAV2)	CLIC-GEEC endocytosis (Nonnenmacher and Weber, 2011)
*Picornaviridae*	Coxsackie virus A9 (CVA9)	Concentration in rafts of the receptor αVβ3-integrin, the coreceptor glucose-regulated protein 78 (GRP78) and MHC-I. Activation of Raf/MAPK signaling pathway in rafts (Triantafilou and Triantafilou, 2003).
	Coxsackie virus B3 (CVB3) RD strain	Receptor GPI-anchored decay accelerating factor (DAF) on rafts (Legler et al., 2005; Coyne and Bergelson, 2006).
	Coxsackie virus B4 (CVB4)	Receptor GPI-anchored decay accelerating factor (DAF) on rafts. Recruitment of the Coxsackie-adenovirus-receptor protein (CAR) into rafts (Triantafilou and Triantafilou, 2004).
	Echovirus type 1 (EV1)	Caveolae-mediated endocytosis (Marjomäki et al., 2002; Pietiäinen et al., 2004). Receptor α2β1-integrin on rafts (Upla et al., 2004).
	Echovirus type 11 (EV11)	Receptor GPI-anchored decay accelerating factor (DAF) on raft (Stuart et al., 2002).
	Enterovirus 71 (EV71)	Raft-dependent activation of PI3K/Akt signaling pathway. Interaction of viral capsid protein VP1 with the receptor SCARB2 within rafts (Zhu et al., 2015). Caveolae-mediated endocytosis via the receptor P-selectin glycoprotein ligand-1 (PSGL-1) (Lin et al., 2013).
	Enterovirus D68 (EV-D68)	Recruitment of viral particles and ICAM-5 receptor into rafts (Jiang et al., 2020).
	Foot-and-mouth disease virus (FMDV)	Caveolae-mediated endocytosis by heparan sulfate binding (O’Donnell et al., 2008).
	Rhinovirus	Receptor intercellular adhesion molecule-1 (ICAM-1) on rafts (Greve et al., 1989; Bacsó et al., 2002).
*Reoviridae*	Avian reovirus (ARV)	Caveolae-mediated endocytosis (Huang et al., 2011; Wang Y. et al., 2020).
	Grass carp reovirus (GCRV)	Caveolae-mediated endocytosis (Zhang et al., 2018a).
	Muscovy duck reovirus (MDRV)	Caveolae-mediated endocytosis (Li et al., 2020a).
	Porcine rotavirus (PRV)	Raft-dependent entry (Dou et al., 2018)
	Rotavirus (RV)	Receptors ganglioside GM1, integrin subunits α2 and β3, heat shock cognate protein Hsc70 on rafts. Association of viral particles with rafts (Arias et al., 2002; Iša et al., 2004; Lopez and Arias, 2006; Guerrero and Moreno, 2012).
*Polyomaviridae*	BK polyomavirus (BKV)	Caveolae-mediated endocytosis (Eash et al., 2004; Eash and Atwood, 2005; Dugan et al., 2006; Moriyama et al., 2007).
	Murine polyomavirus (MuPyV)	Caveolae-mediated endocytosis (Richterová et al., 2001; Gilbert and Benjamin, 2004; Liebl et al., 2006).
	Simian virus 40 (SV40)	Caveolae-mediated endocytosis (Toscano and de Haan, 2018; Chen et al., 2019b). Receptor MHC-I (Stang et al., 1997; Anderson et al., 1998; Norkin, 1999; Parton and Lindsay, 1999), ganglioside GM1 (Tsai et al., 2003; Szklarczyk et al., 2013) on rafts. Non-clathrin non-caveolae endocytosis (Damm et al., 2005).

## Composition of Membrane Rafts

Singer and Nicolson (1972) proposed one of the earliest models to accurately describe the structure of biological membranes, the “fluid mosaic model” of membranes. This model describes the cellular membrane as a uniform lipid bilayer with randomly distributed proteins. However, from the start it was observed that membranes are not uniform, since they are formed by clusters of lipids (Lee et al., 1974). The term “lipid domain” was established in 1982 by Karnovsky et al. (1982), who found that lipids have no homogeneity in their lateral distribution and that such organizational heterogeneity may have functional and structural significance.

A consensus definition for a membrane raft (Figure 1) was established at the 2006 Keystone Symposium of Lipid Rafts and Cell Function: “*Membrane rafts are small (10–200 nm), heterogenous, highly dynamic, sterol- and sphingolipid-enriched domains that compartmentalize cellular processes. Small rafts can sometimes be stabilized to form larger platforms through protein-protein and protein-lipid interactions*” (Pike, 2006). For membrane rafts, the most important interactions are between sterols, saturated phospholipids and sphingolipids, such as glycolipids and sphingomyelin (SM). Cholesterol and saturated lipids interact more strongly with each other than with unsaturated lipids. Because of this, in the presence of cholesterol, sphingolipids are condensed in a unique state of matter called ‘liquid ordered (L_o_) phase’ (Figure 1). In this phase, lipid molecules have a high capacity for lateral diffusion, whereas the surrounding unsaturated lipids form the “liquid disordered (L_d_) phase,” in which lipid molecules are mostly immobile (Levental et al., 2020). Two types of proteins are suggested to be associated with membrane rafts: glycosylphosphatidylinositol (GPI)-anchored proteins and lipidated -specifically palmitoylated- proteins (Sezgin et al., 2017). The raft affinity of transmembrane proteins is dependent on the physicochemical features -palmitoylation, length and surface area- of the transmembrane domains (TMDs), but generally tend to be excluded from lipid rafts (Lorent et al., 2017) (Figure 1).

It is important to keep in mind that most of the analyzed data are based on “detergent-resistant membranes” (DRMs) or “detergent-insoluble glycolipid-enriched membranes” (DIGs). The tight packing of the domains enriched in sphingolipids and cholesterol confers resistance to solubilization with non-ionic detergents (e.g., Triton X-100) at low temperatures (Graham, 2002; Chamberlain, 2004). Due to this property, membrane rafts were originally referred to as DRMs, which are enriched in sterols, sphingolipids and lipidated proteins, but are not exactly the same as membrane rafts. DRMs only exist after detergent treatment and may not necessarily correspond precisely with the native membrane structure present in live cells. The composition and properties of DRMs are dependent on the nature and concentration of the detergent, as well as the temperature and time of solubilization. On the other hand, membrane rafts are *in vivo* transient membrane microdomains, whose existence is independent from the use of detergents (Lichtenberg et al., 2005; Brown, 2006).

## Dependence on Cholesterol or Sphingomyelin Does Not Imply Participation of Membrane Rafts in Viral Entry

The involvement of membrane rafts in viral entry is usually evaluated by the effects of raft-disrupting treatments, which mainly remove cholesterol from the plasma membrane or inhibit its synthesis. Inhibition of virus infection by cholesterol depletion is generally recoverable by the addition of exogenous cholesterol. The most commonly used raft-disrupting compound is methyl-β-cyclodextrin (MβCD), which extracts cholesterol from cells and rafts, abolishing association of raft proteins with DRMs and disrupting raft-regulated cell signaling pathways. However, MβCD not only extracts cholesterol from the plasma membrane, but also from intracellular compartments, thereby disrupting organelle function and structure as well as vesicular transport (Bieberich, 2018). Also, even though “raft-dependent pathways” are highly sensitive to cholesterol-depleting agents, clathrin-mediated endocytosis is also affected at high doses of MβCD (Rodal et al., 1999; Subtil et al., 1999). Thus, it is important to consider that cholesterol dependence may not necessarily prove the participation of intact rafts in viral entry.

Although less common, another experimental tool to analyze the importance of rafts in viral entry is the treatment of membranes with sphingomyelinases (SMases). SMases catalyze the hydrolysis of SM into ceramide, converting rafts into ceramide-rich platforms (CRPs). Although CRPs can be found in DRMs, ceramide no longer forms part of a raft with L_o_ structure, but rather forms its own lipid microdomain structure (Bieberich, 2018). Depletion of SM impairs the entry of, among others, pseudorabies virus (PRV) (Pastenkos et al., 2018), rubella virus (RuV) (Otsuki et al., 2017), Influenza virus A (IAV) (Audi et al., 2020), and hepatitis C virus (HCV) (Voisset et al., 2008) (Table 1). However, as happens with cholesterol, the requirement of SM for viral entry may not imply the participation of membrane rafts *per se*. For instance, the fusion peptide of Classical Swine Fever Virus (SFV) has a high affinity for cholesterol- and sphingolipid-enriched microdomains (Ahn et al., 2002), but using vesicles prepared from synthetic sphingolipids and sterols, it has been demonstrated that membrane rafts are not essential for the virus entry by fusion (Waarts et al., 2002). Throughout the review, we will find more examples about the use of cholesterol- and SM-depleting agents and the subsequent interpretation of the obtained results.

## Membrane Rafts in Virus Attachment

Viral entry into a host cell is a complex process that first requires virus binding to the cell surface, often via a receptor. Viruses interact with different cell surface molecules that comprise a wide variety of proteins, lipids and glycans (Jolly and Sattentau, 2013; Bhella, 2015). The same cell receptor types can be recognized by different viruses, and one virus may be able to interact with more than one cell surface molecule. In some cases, interaction with a single receptor is sufficient to trigger infection, whereas in others it is necessary for the virus to interact with several receptor molecules (Sieczkarski and Whittaker, 2004).

The concentration of receptors, co-receptors and viral particles into rafts promotes activation of cell signaling pathways and enhances the efficiency of the entry process (Szklarczyk et al., 2013). One example is Coxsackie virus A9 (CVA9) infection (Table 2). The concentration of the receptor αvβ3, coreceptor GRP78 and MHC class I (which facilitates virus internalization) is increased within membrane rafts compared to uninfected controls. The production of molecules belonging to the Raf/MAPK pathway also increases during CVA9 infection. In this way, the entry and signaling machinery of the virus is concentrated in membrane rafts, which facilitates efficient viral entry (Triantafilou and Triantafilou, 2003).

Some cellular receptors and co-receptors are constitutively expressed in rafts, such as angiotensin-converting enzyme 2 (ACE2), receptor for severe acute respiratory syndrome coronavirus (SARS-CoV) (Glende et al., 2008; Lu et al., 2008) and SARS-CoV-2 (Hoffmann et al., 2020; Wang H. et al., 2020) (Table 1). In other cases, cell receptors and co-receptors are not constitutively located into rafts, but viral attachment to the cell surface triggers translocation to them. Such relocation of receptors and co-receptors into rafts has been observed during infection by human herpesvirus type 6 (HHV-6) (Tang et al., 2008), Kaposi’s sarcoma-associated herpesvirus (KSHV) (Chakraborty et al., 2011), vaccinia virus (Izmailyan et al., 2012; Schroeder et al., 2012), HIV-1 (Popik et al., 2002; Kamiyama et al., 2009) (Table 1), Coxsackie virus B4 (CVB4) (Triantafilou and Triantafilou, 2004) and enterovirus D68 (EV-D68) (Jiang et al., 2020) (Table 2). However, receptor recruitment into rafts is not always a consequence of a viral infection, although it exerts influence on virus entry. For example, translocation of the herpes simplex virus type 1 (HSV-1) receptor nectin-1 into rafts is induced by the presence of αVβ3-integrin at the plasma membrane, not by HSV-1 attachment to the cell surface (Gianni and Campadelli-Fiume, 2012) (Table 1).

The presence of viral particles and/or viral glycoproteins in membrane rafts during virus attachment is another way to demonstrate the involvement of rafts in entry process. For instance, a fraction of the glycoprotein gB of HSV-1 is associated with cell rafts from the moment of attachment and during entry (Bender et al., 2003) (Table 1). In some infections, such HIV-1 (Popik et al., 2002), HHV-6 (Tang et al., 2008) (Table 1) or echovirus type 71 (EV71) (Zhu et al., 2015) (Table 2), even the interaction of viral glycoprotein with the respective receptor within rafts have been shown. These studies strongly support a role for intact rafts in virus attachment, beyond the use of raft-disrupting agents.

However, although location of viral particles and/or cell receptors on membrane rafts gives us a lot of information about the entry pathway, it is important to consider that viruses may enter through a region different from the initial attachment site. Virus particles can bind to non-raft membranes, but then shift to raft domains. Vaccinia virus particles bind initially to glycosaminoglycans and laminin in non-raft domains, inducing further interactions with integrin β1 within rafts. The subsequent recruitment of the receptor CD98 into rafts and activation of downstream kinases lead to endocytosis of the virus (Izmailyan et al., 2012; Schroeder et al., 2012). In other cases, although viruses initially bind to a raft domain, they shift to another membrane domain for entry. For example, amphotropic murine leukemia virus (A-MLV) binds to large rafts without the protein caveolin-1, but then is transported into caveolae to enter the host cells (Beer and Pedersen, 2006) (Table 1).

## Membrane Rafts in Endocytosis

Endocytosis is a cellular mechanism by which cells internalize substances from the external environment and can be classified into phagocytosis or pinocytosis. Phagocytosis is generally restricted to specialized cells, such as macrophages, and is typically employed to digest bacteria and/or large particles. Pinocytosis is a non-specific, non-saturable, and non-carrier-mediated form of membrane transport via vesicular uptake of fluids, macromolecules and small pathogens (Basturea, 2019). This last endocytic pathway, in turn, has been differentiated based on the coat proteins of the endocytic vesicle into: clathrin-mediated endocytosis and clathrin-independent endocytosis (Basturea, 2019).

Clathrin-mediated endocytosis (CME) (Kaksonen and Roux, 2018) is, to date, the most frequently known mechanism for endocytosis of small and medium-size viruses. After a period of lateral movement along the cell surface, receptor-bound virus particles enter into preexisting clathrin-coated pits (CCPs), which pinch off from the plasma membrane to form clathrin-coated vesicles (CCVs) that subsequently lose their clathrin coat and fuse with endosomes. This mechanism is dependent on dynamin-II, a GTPase required for detachment of coated vesicles from the plasma membrane (Singh et al., 2017).

Clathrin-independent endocytosis (CIE) pathways (Mayor et al., 2014; Sandvig et al., 2018; Shafaq-Zadah et al., 2020) are cholesterol-sensitive mechanisms that may be classified into caveolae-mediated endocytosis (dynamin-dependent) and non-clathrin non-caveolae mediated endocytosis, which can be dynamin-dependent (VanHamme et al., 2008) or independent (Damm et al., 2005). Caveolae (Bastiani and Parton, 2010; Cheng and Nichols, 2016) are the best characterized microdomains of membrane rafts, which consist of small plasma membrane invaginations at the surface of several mammalian cell types. They can bud into cells in the form of endocytic vesicles that merge to early endosomes for cargo delivery (Pelkmans et al., 2004). The inner layer of the caveolar coat is composed of caveolins, proteins with a hydrophobic transmembrane loop inside the membrane and both N- and C- termini facing the cytoplasm. There are three types of caveolins, Cav1, Cav2, and Cav3, the former being the major component of caveolae (Williams and Lisanti, 2004). Cav1 is a palmitoylated protein which uses cholesterol for binding to rafts and can influence numerous cellular processes by forming oligomers that interact with signaling molecules, regulate the cholesterol content of caveolae and lead to the formation of complex scaffold domains implied in caveolae biogenesis (Khater et al., 2019). On the other hand, the outer layer of the caveolar coat is composed by cavins (Briand et al., 2011), proteins that constitute homo- and hetero-oligomeric complexes which are thought to stabilize the caveolin scaffold and promote membrane curvature and budding of caveolae (Bastiani et al., 2009; McMahon et al., 2009; Kovtun et al., 2015).

In 2001, it was suggested that caveolar vesicles fuse with a newly discovered organelle called a “caveosome” (Pelkmans et al., 2001), characterized by a neutral pH and the presence of Cav1. However, in 2010 the same authors (Hayer et al., 2010a) clarified that caveosomes actually correspond to late endosomes modified by the accumulation of the overexpressed Cav1 awaiting degradation (Hayer et al., 2010b). Because of this, authors recommended that the term should no longer be used.

More recently, an alternative classification system has been established, taking into consideration the role of lipid rafts in endocytosis. Depending on membrane rafts, endocytic pathways may be classified into (i) pathways in which lipid rafts are not present in the endocytic vesicle -CME-; (ii) pathways for which endocytic vesicle can contain rafts and non-raft domains -phagocytosis and macropinocytosis-; and (iii) pathways that take place in membrane rafts – the majority of CIE-. The endocytic vesicles that are formed in lipid rafts can be stabilized by the enrichment of certain proteins, such as caveolin (caveolae-mediated endocytosis) or flotillin (flotillin-dependent endocytosis). Alternatively, endocytic vesicles can be formed into lipid rafts by the action of small guanosine triphosphatases (GTPasas), such as Cdc42 and Arf1 (GRAF1-dependent endocytosis), Arf6 (Arf6-dependent endocytosis) or RhoA (RhoA-dependent endocytosis) (El-Sayed and Harashima, 2013).

### Viral Entry by Vesicle Formation in Membrane Rafts

Cell endocytic mechanisms can be exploited by viruses to enter host cells (Barrow et al., 2013; Cossart and Helenius, 2014). Receptor-virus attachment facilitate the concentration of viral particles and/or the activation of signaling pathways, promoting the membrane curvature and, as a consequence, viral endocytosis (Grove and Marsh, 2011). Subsequent penetration into the cytosol generally occurs through early or late endosomes, although additional penetration sites, such as the endoplasmic reticulum, are possible (Yamauchi and Helenius, 2013). The majority of both enveloped and naked viruses require a decrease in the pH of endocytic organelles in order to activate viral surface proteins involved in escape into the cytoplasm, prior to arrival in the degradative lysosome (Nicola, 2016).

**FIGURE 2:**
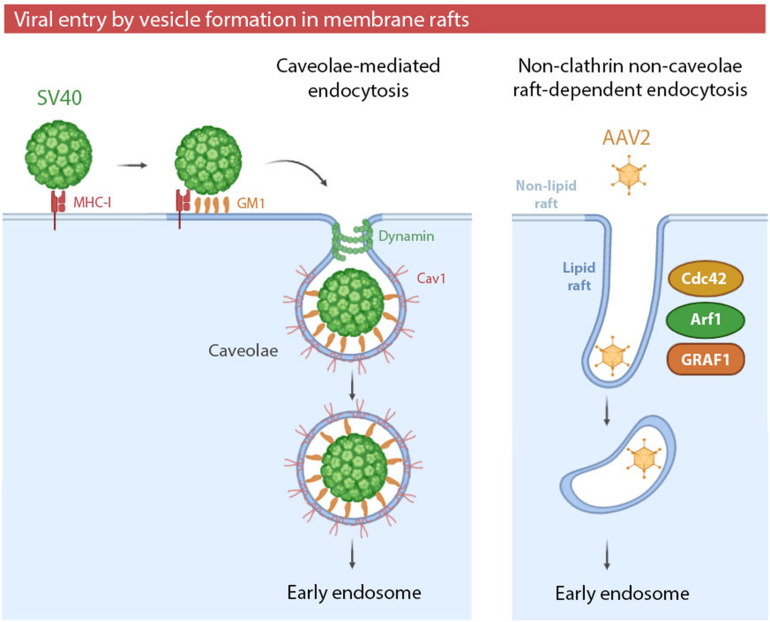
(i) Simian virus 40 (SV40) entry by caveolae. SV40 binding to major histocompatibility class I (MHC-I) molecules targets the viral particles to lipid rafts, where multivalent binding to the ganglioside GM1 promotes viral entry by caveolae. Caveolae-mediated endocytosis can be exploited by other viruses, such as HCoC-229E, JEV or EV71. (ii) Adeno-associated virus type 2 (AAV2) entry by non-clathrin non-caveolae raft-dependent endocytosis. AAV2 enters the cells via clathrin-independent carrier/GPI-anchored protein early endosomal compartment (CLIC/GEEC). The formation of tubular vesicles by GRAF1-dependent endocytosis is mediated by the complementary roles of Cdc42 and Arf1 in regulating actin polymerization. Non-clathrin non-caveolae raft-dependent endocytosis can be also exploited by SV40 and SARS-CoV.

Viral entry via endocytic vesicles can occur in raft domains of the plasma membrane. These raft-dependent pathways can be caveolae-mediated or clathrin- and caveolae- independent. Whereas caveolae-mediated endocytosis has been highly studied and reviewed in both enveloped and non-enveloped viruses (Xing et al., 2020), non-clathrin non-caveolae raft-dependent endocytosis is still poorly characterized. This novel mechanism was described for the first time in 2005 as an alternative entry pathway for Simian virus 40 (SV40) (Damm et al., 2005) (Table 2). Until 2005, it was believed that SV40 entered cells only by caveolae-mediated endocytosis (Figure 2). SV40 entry by caveolae has been extensively studied since 1996 (Anderson et al., 1996) and the entry of this virus is used as a control of caveolae-mediated endocytosis in several studies (Quirin et al., 2008). The new alternative pathway proposed for SV40 was not only caveolae independent, but also independent of clathrin, dynamin-II and Arf6 (a small GTPase involved in recycling pathway). However, it was cholesterol-sensitive and tyrosine kinase dependent. Thus, viral particles were associated with DRMs during the early stages of the viral cycle, revealing an involvement of intact lipid rafts (Damm et al., 2005).

More recently, viral entry mediated by non-clathrin non-caveolae endocytosis in lipid rafts has been especially characterized in adeno-associated virus 2 (AAV2) (Table 2) infection (Figure 2). AAV2 particles enter host cells via GRAF1-dependent endocytosis, also known as CLIC/GEEC (clathrin-independent carrier/GPI-anchored protein early endosomal compartment) (Nonnenmacher and Weber, 2011). GEECs are vesicles derived from membrane rafts whose formation is independent of clathrin, caveolae, dynamin and Rac1. GEECs are formed from fusion of smaller CLICs. Actin polymerization -regulated by Cdc42 and Arf1- and GRAF1 protein drives the initial formation of CLICs and, consequently, GEEC generation (Shafaq-Zadah et al., 2020). The role of membrane rafts in viral entry by CLIC/GEEC has been proven by use of cholesterol-depleting reagents, the recovery of AAV2 from isolated membrane fractions enriched in raft markers, and by the physical association of virions and GRAF1-enriched vesicles in lipid rafts (Nonnenmacher and Weber, 2011).

In viruses such as human adenovirus species C (HAdv-C) (Colin et al., 2005), rhesus rotavirus (RRV) (Sánchez-San Martín et al., 2004) and EV1 (Krieger et al., 2013) the presence of non-clathrin non-caveolae cholesterol-sensitive endocytosis has been observed but, in contrast to SV40 or AAV2 entry, it has not been demonstrated to be raft-dependent. The non-clathrin non-caveolae pathway is especially controversial since it is sensitive to cholesterol depletion, but decreased viral entry caused by cholesterol-depleting reagents does not necessarily mean lipid raft involvement. One of the best examples of a virus that enters by a non-clathrin non-caveolae endocytosis which is cholesterol-sensitive but raft-independent is lymphocytic choriomeningitis virus (LCMV). Although cholesterol depletion with MβCD produced a reduction in LCMV infection (Rojek et al., 2008), the same MβCD concentrations they used inhibited several plasma membrane processes, including CME. After adjusting the concentration of raft-disrupting reagents to a lower level, sufficient to block the infection of the raft-dependent SV40 but without effects on CME of SFV, LCMV infection was no longer inhibited. Therefore, the authors proposed that LCMV entry is cholesterol-dependent but raft-independent (Quirin et al., 2008). A possible cause for this cholesterol sensitivity could be the association between the LCMV receptor – dystroglycan (DG) – with non-raft cholesterol, which is critical for LCMV infection (Shah et al., 2006).

Another example of raft-independent entry is the non-clathrin non-caveolae endocytosis of HIV-1 in polarized trophoblastic cells. Treatment with the cholesterol-sequestering drug filipin severely impaired virus internalization, whereas treatment with MβCD had no impact on this pathway. Part of the reduction in HIV-1 infectivity in the presence of filipin may be related to an indirect effect of the drug on HIV-1 gene expression. Collectively, authors concluded that the pathway requires free membrane cholesterol, and that membrane rafts appear to be involved at later points of virus entry process (Vidricaire and Tremblay, 2007).

### Membrane Rafts in Viral Entry via Macropinocytosis

Macropinocytosis is a specialized form of CIE that is dependent upon cortical actin ruffling and results in the internalization of large amounts of fluid by enlarged vesicles, denominated macropinosomes (Donaldson, 2019). Regulatory factors of macropinocytosis include PAK-1, Arf6 and the Rho family GTPases, Rac1 and Cdc42. The main difference between phagocytosis and CME is dynamin-II independence (Mercer and Helenius, 2009). A relationship between macropinocytosis and lipid rafts has not been completely elucidated but cannot be ruled out. Macropinocytosis is sensitive to cholesterol-depletion and membrane ruffling may occur in raft domains. On the other hand, macropinosome formation requires vast areas of the plasma membrane. As a consequence, the final vesicle can be formed by a mixture of raft and non-raft domains (El-Sayed and Harashima, 2013).

Viruses can activate the signaling pathways that trigger macropinocytosis, promoting actin-mediated membrane ruffling and blebbing and the resulting macropinosome formation. Macropinocytosis has been linked to a requirement of cholesterol for viral entry. For instance, the entry of HIV-1 in the brain microvascular endothelia takes place by macropinocytosis, and is inhibited by cholesterol-extracting agents (Liu et al., 2002). As discussed before, a cholesterol requirement does not imply the involvement of rafts, but in certain infections by enveloped viruses a close relationship between macropinocytosis and membrane rafts *per se* has been demonstrated. For example, in Ebola virus (EBOV) infection (Table 1). Using fluorescently labeled Ebola virus like particles (VLPs), it is possible to visualize the dynamic internalization into live cells by macropinocytosis through membrane rafts (Jin et al., 2020).

**FIGURE 3:**
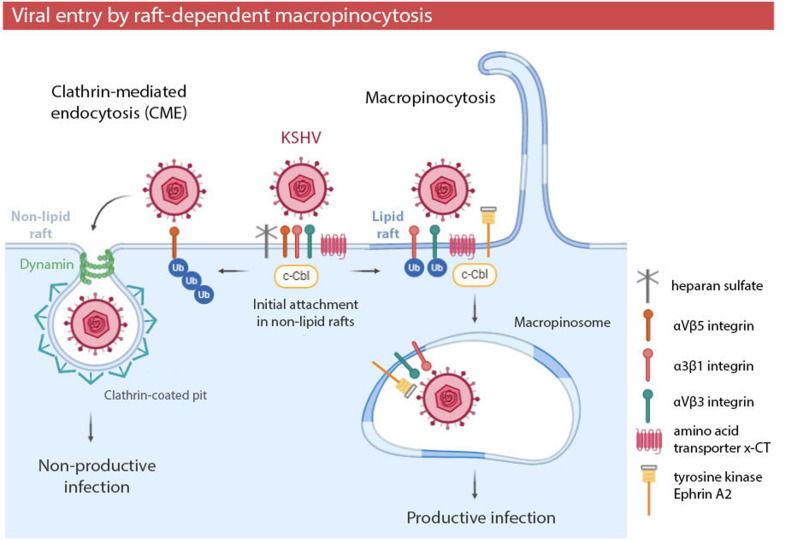
Kaposi’s sarcoma-associated herpesvirus (KSHV) entry by raft-dependent macropinocytosis in endothelial cells. Initial viral attachment occurs in non-lipid rafts. E3 ubiquitin ligase c-Cbl and membrane rafts determine the posterior location of receptors and the KSHV entry pathway. Polyubiquitination of the receptor αVβ5 leads to non-productive viral entry via clathrin in non-lipid rafts. Monoubiquitination of αVβ3 and α3β1 integrins induces the translocation of viral particles and receptors to lipid rafts, leading to productive viral entry via macropinocytosis. Viral entry by macropinocytosis in a raft-dependent manner has been also observed in EBOV infection.

The involvement of rafts in macropinocytosis has been especially studied in KSHV infection (Table 1 and Figure 3). Cholesterol depletion decreases KSHV infection and viral gene expression, suggesting that lipid rafts play a role in the entry and post binding stage (Raghu et al., 2007). Further research showed that the initial attachment of KSHV with heparan sulfate, α3β1, αVβ3, and αVβ5 integrins and the amino acid transporter x-CT occurs in non-raft domains (Veettil et al., 2014). The interaction of KSHV with these receptors induces the recruitment of the adaptor c-Cbl (Veetti et al., 2010), which is an E3 ubiquitin ligase which is able to influence cellular signal pathways by ubiquitinating target proteins to control their localization. c-Cbl selectively monoubiquitinates α3β1 and αVβ3 integrins, promoting the translocation of viral particles and receptors into membrane rafts at the junctional base of macropinocytic blebs (Chakraborty et al., 2011). In these raft domains, KSHV interacts with tyrosine kinase Ephrin A2 (EphA2) to amplify cascade signaling and to promote macropinosome formation (Chakraborty et al., 2012; Hahn et al., 2012). On the other hand, c-Cbl may polyubiquitinate αVβ5 integrin in non-lipid rafts, preventing its translocation into raft domains and inducing non-productive viral entry via clathrin (Chakraborty et al., 2011).

These results show the relevance of membrane rafts in entry by macropinocytosis of certain viruses. Lipid rafts not only may constitute part of the vast area of plasma membrane required for macropinosome formation, but also could allow the clustering of viral particles and receptors and, as a consequence, the recruitment of signaling molecules necessary for macropinocytosis induction.

### Relationship Between Membrane Rafts and Viral Entry by Clathrin

Initially, it seemed clear that CME was raft-independent. Some studies suggested the absence of rafts in CCPs (Nichols, 2003b), and there were general observations that rafts and raft-associated proteins internalize through CIE (Sato et al., 2004). However, membrane rafts can cooperate with clathrin in the internalization of some molecules, such as the B cell antigen receptor (BCR) (Stoddart et al., 2002) or certain GPI-anchored proteins (Rollason et al., 2007; Sarnataro et al., 2009).

Regarding viral infection, there are studies showing a dependence on both clathrin and cholesterol for cell entry, as in the case of varicella-zoster virus (VZV) (Hambleton et al., 2007), type C food-and-mouth- disease virus (FMDV) (Martín-Acebes et al., 2007), Crimea-Congo hemorrhagic fever virus (CCHFV) (Simon et al., 2009), Japanese encephalitis virus (JEV) (Yang et al., 2013) and HIV-1 (Praena et al., 2020). Besides, viruses internalized by clathrin may require cholesterol and/or SM to escape from the endocytic vesicle via fusion. This is the case of Semliki Forest virus (SFV) and Sindbis virus (SIN) (Phalen and Kielian, 1991; Nieva et al., 1994; Marquardt and Kielian, 1996; Smit et al., 1999). However, a cholesterol or SM requirement does not imply a direct relation between clathrin and membrane rafts *per se*.

The implication of intact membrane rafts in CME actually occurs at the virus attachment step. For example, cell surface attachment in hepatitis C virus (HCV) -via tetraspanin CD81 and scavenger receptor B type I (SR-BI) (Kapadia et al., 2007) - and the attachment of infectious bronchitis virus (IBV) (Guo et al., 2017) occurs in raft domains. However, after virus binding, entry of both HCV (Blanchard et al., 2006; Meertens et al., 2006; Helle and Dubuisson, 2008) and IBV (Wang et al., 2019a, b) are mediated by clathrin. One of the best examples of the possible relation between rafts and clathrin is the entry of JEV in neural stem cells. JEV infection is inhibited by cholesterol depletion, and viral envelope proteins (glycoprotein E and Nakayama) are associated with rafts in the early stage of infection. Glycoprotein E colocalizes with cholera toxin B (CTB), which enters cells in a raft-dependent manner, but can also be internalized by CME (Chinnapen et al., 2007; Day and Kenworthy, 2015). The possibility of caveolae-mediated endocytosis was raised, but the glycoprotein did not colocalize with Cav1. However, it colocalizes with transferrin – which is trafficked via clathrin (Mayle et al., 2012) – and clathrin-null mutants had reduced infection. Also, rafts are required for activation of the phosphoinositide 3-kinase/Akt signaling pathway in the early stage of infection (Das et al., 2010). For initiation of this cascade, recruitment of the receptor HSP70 into rafts is necessary (Zhu et al., 2012b). All these results suggest the possible involvement of rafts as a platform to concentrate JEV particles and their cellular receptors, and the subsequent virus internalization by CCPs.

On the other hand, there is emerging evidence that some viruses take advantage of cross-talk between clathrin- and caveolae-mediated pathways. Specifically, there are studies that propose that JC virus (JCV) (Querbes et al., 2006), bovine papillomavirus type 1 (BPV1) (Laniosz et al., 2008) and human papillomavirus type 16 (HPV-16) (Laniosz et al., 2009) enter cells via CCPs which then require Cav1-mediated trafficking for infection.

## Membrane Rafts in Viral Entry by Fusion

Fusion process is a crucial entry mechanism for certain enveloped viruses. Viral fusion proteins are stimulated by a signal during the attachment at the target cell -such as receptor or co-receptor binding or proton binding in an endosome- promoting a series of conformational changes. The hydrophobic segment known as “fusion peptide” triggers the viral-cell membrane fusion, process which requires cooperation between lipids and proteins (Rawat et al., 2003; Harrison, 2015; Barrett and Dutch, 2020). Viral proteins play an essential role in directing and catalyzing the process, but successful outcome may depend on the lipid composition of both viral and cell membranes. Although the role of rafts in viral entry by fusion has been examined recently (Risselada, 2017), the mechanisms by which dynamic raft components control the process remain unclear.

Several studies have shown the importance of cholesterol for viral fusion processes, not only in the cell plasma membrane (or endosomal membrane), but also within the viral envelope. This is the case of HIV-1 (Mañes et al., 2000; Guyader et al., 2002), ecotropic murine leukemia virus (E-MLV) (Lu et al., 2002), VZV (Hambleton et al., 2007), human parainfluenza virus type 3 (HPIV3) (Tang et al., 2019) and caprine parainfluenza virus type3 (CPIV3) (Li et al., 2020b). There are also studies that have implicated sphingolipids in fusion events. In HIV-1 infection (Table 1), glycosphingolipids such as globotriaosyl ceramide (Gb3) (Puri et al., 1998), GM3 ganglioside and galactosylceramide (GalCer) (Hammache et al., 1998a, b) interact with viral glycoproteins and are suggested to act as fusion cofactors, promoting receptor recruitment and clustering (Sáez-Cirión et al., 2002; Rawat et al., 2004, 2006; Viard et al., 2004). In SFV infection, SM has also been proposed to act as a fusion cofactor, possibly activating the viral fusion protein in a specific manner (Nieva et al., 1994).

**FIGURE 4:**
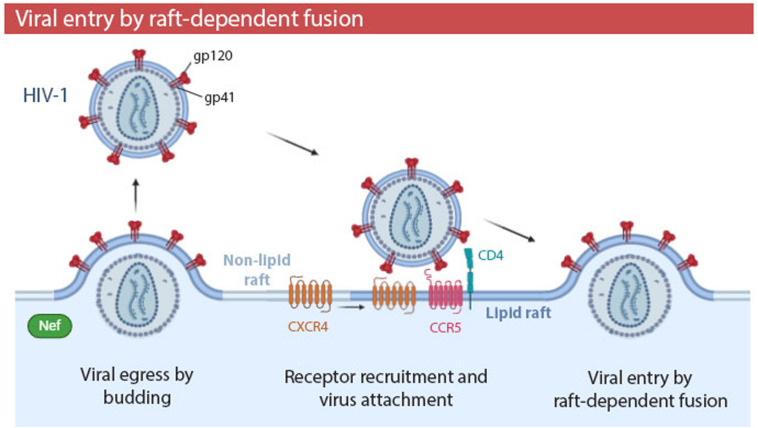
Human immunodeficiency virus type 1 (HIV-1) entry by raft-dependent fusion in T lymphocytes. HIV-1 binds to CD4 receptor and CCR5 co-receptor within rafts. Interaction of CD4 with gp120 induces conformation changes which recruit the CXCR4 co-receptor to the periphery of the raft. Fusogenic activity of gp41 promotes fusion of the viral envelope and plasma membrane. The HIV-1 virulence factor Nef promotes budding of HIV-1 from membrane rafts, generating a viral progeny with a higher proportion of rafts in their envelopes and, thus, a higher infectivity. Viral entry by fusion in a raft-dependent manner has been also observed in EBOV, HSV-1, and MHV infections.

The relevance of cholesterol and SM is not surprising, considering that several viral families, including *Orthomyxoviridae*, *Paramyxoviridae*, *Filoviridae*, and *Retroviridae* (Manié et al., 2000; Suomalainen, 2002) use membrane rafts as a budding site to egress the cell and, therefore, their viral envelopes are cholesterol- and sphingolipid- enriched. Lipid rafts and viral envelopes are not only similar in their lipid composition, but also in their protein composition. In both structures, transmembrane proteins are enriched in palmitoylated and phosphorylated residues, although the TMDs of viral proteins are generally shorter and have a smaller accessible surface area per residue. Viral envelope proteins differ from raft proteins in other posttranslational modifications. For example, viral proteins have shorter myristoyl residues and higher phosphorylation in terms of protein fraction (Yurtsever and Lorent, 2020). Budding site selection is a fundamental step in the life cycle of enveloped viruses, as it determines the lipid and protein composition of their envelopes, which will influence the future stability and infectivity of virions. In HIV-1 infections (Table 1), the virulence factor Nef promotes HIV-1 budding from lipid rafts (Figure 4). Consequently, in the presence of Nef, viral envelopes contain more ganglioside GM1 -a major component of rafts- and infectivity of HIV-1 is significantly increased (Zheng et al., 2001; Mukhamedova et al., 2019).

To demonstrate the implication of rafts in the fusion process, some studies have analyzed the association of viral fusion proteins with cell membrane rafts, with the GP2 viral fusion protein of EBOV being one example (Freitas et al., 2011) (Table 1). Other studies have proven the presence of receptors necessary for fusion within lipid rafts, as is the case of HIV-1 infection (Table 1 and Figure 4). CD4 receptor (Millán et al., 1999; Kozak et al., 2002) and CCR5 co-receptor of HIV-1 (Popik et al., 2002) are located in raft domains of T lymphocytes. The CXCR4 co-receptor is almost absent on rafts, but the interaction of CD4 with viral glycoprotein gp120 induces conformational changes which recruit CXCR4 to the periphery of the raft (Popik et al., 2002; Kamiyama et al., 2009). Cholesterol has been proposed to organize this multimeric envelope/receptor complex in clusters (Sáez-Cirión et al., 2002; Viard et al., 2002). However, it has been also suggested that the presence of HIV-1 receptors in rafts is not required for fusion process, and that cholesterol modulates the HIV-1 entry independently of its ability to promote raft formation (Percherancier et al., 2003). On the other hand, recent studies have demonstrated that the interfaces between Lo and Ld lipid domains are the predominant sites of HIV-1 fusion. Moreover, the presence of Lo domains in the cell membrane increases the overall fusion kinetics (Yang et al., 2016, 2017). The raft boundary can act as an attractor for viral fusion peptides (Molotkovsky et al., 2018).

The involvement of rafts in fusion process may be very elusive, not only in HIV-1 infection, but also in other viruses. In murine hepatitis virus (MHV) infections, cholesterol is necessary for fusion, even being proposed as an essential fusion membrane cofactor (Thorp and Gallagher, 2004), and the viral spike (fusion) protein is associated with rafts on the plasma membrane (Choi et al., 2005). However, the fusion receptor CEACAM is not located in rafts (Thorp and Gallagher, 2004), MHV does not incorporate rafts into the virion and the attachment step takes places in non-raft regions. It has been proposed that although MHV binding occurs in non-raft regions, the virus shifts to membrane rafts for viral entry (Choi et al., 2005).

## Choice of Viral Raft-Mediated Entry Pathways

Since viruses can exploit more than one entry mechanism, even a combination of raft-dependent and -independent processes is possible. One such example is influenza A virus (IAV) (Table 1), whose main entry mechanism is via clathrin (Lakadamyali et al., 2008), but can also enter by non-clathrin non-caveolae endocytosis (Sieczkarski and Whittaker, 2002; Rust et al., 2004) and macropinocytosis (de Vries et al., 2011). Recently it has been proposed that rafts may play a role in IAV entry, acting as host attachment factors for multivalent binding, possibly through a raft-dependent endocytic pathway (Verma et al., 2018). Another example is EBOV (Table 1), which enters cells mostly by macropinocytosis (Nanbo et al., 2010; Saeed et al., 2010) but also by clathrin- (Bhattacharyya et al., 2010; Aleksandrowicz et al., 2011) and caveolae-mediated endocytosis (Bavari et al., 2002; Empig and Goldsmith, 2002; Sanchez, 2007).

Generally, a specific viral entry pathway prevails over the others. The choice of primary entry mechanism can sometimes be virus strain-specific. For instance, in keratinocytes, human papillomavirus (HPV) type 31 (Table 2) enters via caveolae (Smith et al., 2007), whereas HPV type 16 enters via clathrin (Laniosz et al., 2009). Another example is West Nile virus (WNV) in Vero cells (Table 1); the Sarafend strain binds to αvβ3 integrin and enters by CME (Chu and Ng, 2004a, b), whereas the NY385-99 strain exploits a raft-dependent endocytic route which is not associated with the integrin (Medigeshi et al., 2008). On the other hand, the choice of primary entry mechanism may often depend on the cell type. Though JEV (Table 1) uses a caveolae-mediated endocytosis in neurons (Zhu et al., 2012a; Kalia et al., 2013), it changes to a CME in Vero and Huh7 cells (Nawa et al., 2003; Tani et al., 2010), neural stem cells (Das et al., 2010) and porcine kidney cells (Yang et al., 2013).

In general, the cellular determinants of the route of viral entry are unknown. However, there are studies that have proposed certain cellular factors that determine this choice. For instance, αVβ3-integrin on the cell surface determines which entry pathway is used by HSV-1 (Table 1); in its presence, HSV-1 entry is mediated by cholesterol and dynamin-II, whereas in cells lacking the integrin, HSV-1 entry is independent of both. Also, integrin overexpression may favor the HSV-1 entry by macropinocytosis in certain cells (Gianni et al., 2010; Gianni and Campadelli-Fiume, 2012). The same authors have studied the interaction between different integrins and the HSV-1 glycoproteins gH/gL. Whereas αvβ8-integrin promoted viral endocytosis mediated by cholesterol and dynamin-II, αvβ6-integrin favored an endocytic mechanism independent of both (Gianni et al., 2013). Another example is EV71 infection (Table 2). The human scavenger receptor class B member 2 (hSCARB2) activates CME (Lin et al., 2012), whereas the receptor P-selectin glycoprotein ligand-1 (PSGL-1) initiates EV71 endocytosis mediated by caveolae (Lin et al., 2013).

## Conclusion

The role of membrane rafts in viral entry is not easy to elucidate. Viral entry is a complex process composed of different steps, and raft domains may not be implicated in all of them. In some cases, raft participation has only been demonstrated during the attachment event, without insight about an involvement in endocytosis or fusion. In other cases, there is a clear dependence between rafts and viral endocytosis, but initial virus binding occurs in non-raft domains. These studies become more complicated due to the fact that one virus can hijack multiple entry mechanisms in the same cell. Moreover, entry pathways can change according to viral strain and cell line, and the reasons for the predominance of one entry pathway over the others are not clear most of the time. Finally, it is important to consider that although many studies analyze the effects of cholesterol depletion in viral entry, cholesterol dependence may not necessarily imply the participation of membrane rafts, but rather need more research to draw conclusions. The same precaution should be taken when extrapolating data obtained from DRMs, as well as when using certain entry controls, such as SV40 or cholera toxin B, which have been shown to exploit different endocytic pathways.

Although many questions remain to be answered, current studies have already shown the relevance of membrane rafts as portals for viral entry. Several viruses that hijack membrane rafts to enter the cells are pathogens of public health importance. Knowledge about the entry mechanisms mediated by rafts can help us to understand their life cycles and, as a consequence, may drive forward future discoveries of novel antiviral therapies.

## Author Contributions

IR and RB-M: conceptualization. IR: writing—original draft preparation. IR, RB-M, SA, and JL-G: writing—review and editing. JL-G: project administration. RB-M and JL-G: funding acquisition. All authors have read and agreed to the published version of the manuscript.

## Conflict of Interest

The authors declare that the research was conducted in the absence of any commercial or financial relationships that could be construed as a potential conflict of interest.
